# Universal peptide-based potential vaccine design against canine distemper virus (CDV) using a vaccinomic approach

**DOI:** 10.1038/s41598-024-67781-5

**Published:** 2024-07-18

**Authors:** Santiago Rendon-Marin, Julián Ruíz-Saenz

**Affiliations:** 1 Grupo de Investigación en Ciencias Animales - GRICA, Facultad de Medicina Veterinaria y Zootecnia, Universidad Cooperativa de Colombia, sede Bucaramanga, Bucaramanga, Colombia; 2 Grupo Infettare, Facultad de Medicina, Universidad Cooperativa de Colombia, Medellín, Colombia

**Keywords:** Vaccinomics, Peptide, In silico, *Morbillivirus canis*, Vaccine design, Immunogenic, Vaccines, Virology

## Abstract

Canine distemper virus (CDV) affects many domestic and wild animals. Variations among CDV genome linages could lead to vaccination failure. To date, there are several vaccine alternatives, such as a modified live virus and a recombinant vaccine; however, most of these alternatives are based on the ancestral strain Onderstepoort, which has not been circulating for years. Vaccine failures and the need to update vaccines have been widely discussed, and the development of new vaccine candidates is necessary to reduce circulation and mortality. Current vaccination alternatives cannot be used in wildlife animals due to the lack of safety data for most of the species, in addition to the insufficient immune response against circulating strains worldwide in domestic species. Computational tools, including peptide-based therapies, have become essential for developing new-generation vaccines for diverse models. In this work, a peptide-based vaccine candidate with a peptide library derived from CDV H and F protein consensus sequences was constructed employing computational tools. The molecular docking and dynamics of the selected peptides with canine MHC-I and MHC-II and with TLR-2 and TLR-4 were evaluated. In silico safety was assayed through determination of antigenicity, allergenicity, toxicity potential, and homologous canine peptides. Additionally, in vitro safety was also evaluated through cytotoxicity in cell lines and canine peripheral blood mononuclear cells (cPBMCs) and through a hemolysis potential assay using canine red blood cells. A multiepitope CDV polypeptide was constructed, synthetized, and evaluated in silico and in vitro by employing the most promising peptides for comparison with single CDV immunogenic peptides. Our findings suggest that predicting immunogenic CDV peptides derived from most antigenic CDV proteins could aid in the development of new vaccine candidates, such as multiple single CDV peptides and multiepitope CDV polypeptides, that are safe in vitro and optimized in silico. In vivo studies are being conducted to validate potential vaccines that may be effective in preventing CDV infection in domestic and wild animals.

## Introduction

*Morbillivirus canis,* commonly known as canine distemper virus (CDV) is a single-negative stranded RNA virus belonging to the *Paramyxoviridae* family that naturally infects a vast array of carnivorous and noncarnivorous species including domestic dogs, raccoons, ferrets, and other wildlife animals^1^. It is the causative agent of a highly contagious disease known as canine distemper (CD), which is characterized by respiratory, digestive, skin, and neurological symptoms^2^. CDV has an RNA genome that includes six linearly organized transcription units, which give rise to eight viral proteins, including the hemagglutinin (H) and the fusion (F) protein^3^. H is a glycoprotein that is involved in adhesion and interaction with cellular receptors and has the greatest genetic variation^4^. Moreover, the F protein facilitates viral and host cell membrane fusion, facilitating viral genome entry into the cytoplasm^5^. Both structural proteins are considered the main antigenic determinants of CDV since a greater number of H- and F-derived peptides are recovered from major histocompatibility complex (MHC) molecules, than from other CDV proteins^6^.

To date, there are diverse CDV lineages distributed worldwide^7,8^. CDV has a high genomic substitution rate, where circulating variants differ by more than 10% at the amino acid level from ancestral strains used in vaccines and other circulating strains around the world^7^. This variation could imply consequences in the vaccine-induced immune response, and the constant occurrence of disease even in vaccinated animals^9^, in addition to the worldwide re-emergence of infections in wildlife, for which commercial vaccines cannot be used due to the lack of safety and efficacy data for most susceptible species^3^. There is no specific treatment for the disease and most efforts are focused on prevention by administering two or more doses of a vaccine between the sixth or seventh week of age and up to three or four months, followed by revaccination every three years throughout the life of the animal^10^.

Some alternatives vaccines include a modified live virus (MLV) type vaccine based on the ancestral strain Onderstepoort. Therefore, considering that this variant no longer circulates, the immunity generated by this vaccine may decrease compared to that of other lineages distributed worldwide^11^. There is a recombinant vaccine that uses a backbone of the canarypox virus, which expresses the H and F proteins of CDV, which protects against the development of symptomatic distemper^12^. In addition, a new platform, considered a recombinant bivalent vaccine, uses the rabies virus, which expresses the CDV H and F proteins, and has been tested in domestic dogs and ferrets^13^. Moreover, a replication-competent adenovirus-vectored vaccine has been developed as a single oral immunization in mice^14^. However, they are not available commercially.

The use of computational tools has become an essential key to developing new generation vaccines for diverse models, including peptide-based therapies and mRNA vaccines^15,16^, considering that the development of alternative vaccines based on experimental methods is time-consuming and financially expensive^17^. This field has been called vaccinomic, which refers to the integration of immunogenetics and immunogenomics with systems biology and immune profiling, as well as immunoinformatics^18^. Hence, the application of “omics” technologies has advanced in the field of vaccinology through the characterization of host-vector-pathogen molecular interactions and the identification of potential protective antigens^19,20^. Epitope-based peptide vaccines are based on in-silico prediction of immunogenic peptides from antigenic dominant pathogen proteins^21,22^. The use of peptides derived from viral antigens could allow B cells to be stimulated by helper T cells and become plasma cells to produce antibodies. In addition to neutralization by antibodies, helper CD4+ T cells and CD8+ T cytotoxic cells are required for complete virus clearance from the host^23^. In addition, T-cell-mediated immunity is dependent on MHC–peptide complexes, peptides that come from the antigen. MHC proteins are encoded by the dog leukocyte antigen (DLA). Each of the DLA alleles represents only a specific set of peptides on the surface of an infected cell and is recognized by T-cell receptors^23^. Hence, antigenic peptides could take advantage of immunological processes involving helper CD4+ T cells and CD8+ T cytotoxic cells^17^.

Peptide-based vaccines have been employed for other viral agents such as hepatitis B, influenza A, and hepatitis C, among others, which exhibit certain immunity in employed models^24–26^. There are many reasons to consider peptide-based vaccines since they are not infectious materials. In addition to the easy introduction of molecules to improve immunogenicity, they could be lyophilized preparations, which provides an advantage in storage. Moreover, there is no risk of reversal of virulence, and they can be designed for the inclusion of multiple antigenic determinants. However, no peptide-based vaccine is commercially available^27^.

After all, a research question has arisen. Can we predict and evaluate peptides with immunogenic potential based on the genetic and antigenic information of worldwide circulating variants? To this aim, a peptide library derived from CDV H and F protein consensus sequences from circulating strains worldwide with immunogenic potential through computational tools was built. Additionally, the molecular interactions of the selected peptides with canine MHC-I, MHC-II, and TLR-2 and TLR-4 were evaluated via molecular docking and dynamic simulations. Moreover, the in silico safety of the peptides was assayed through their antigenicity, allergenicity, toxicity potential, and homologous canine peptides, and the in vitro safety was also evaluated through cytotoxicity in cell lines and canine peripheral blood mononuclear cells (cPBMCs) and a hemolysis assay in canine red blood cells. Multiple polypeptide epitopes were constructed, synthetized, and evaluated based on the best single peptides to validate and compare the usage of multiple immunogens to that of a single antigen based on linked immunogenic predicted peptides.

## Results

### Construction of an immunogenic potential peptide library using computational tools

**Figure 1:**
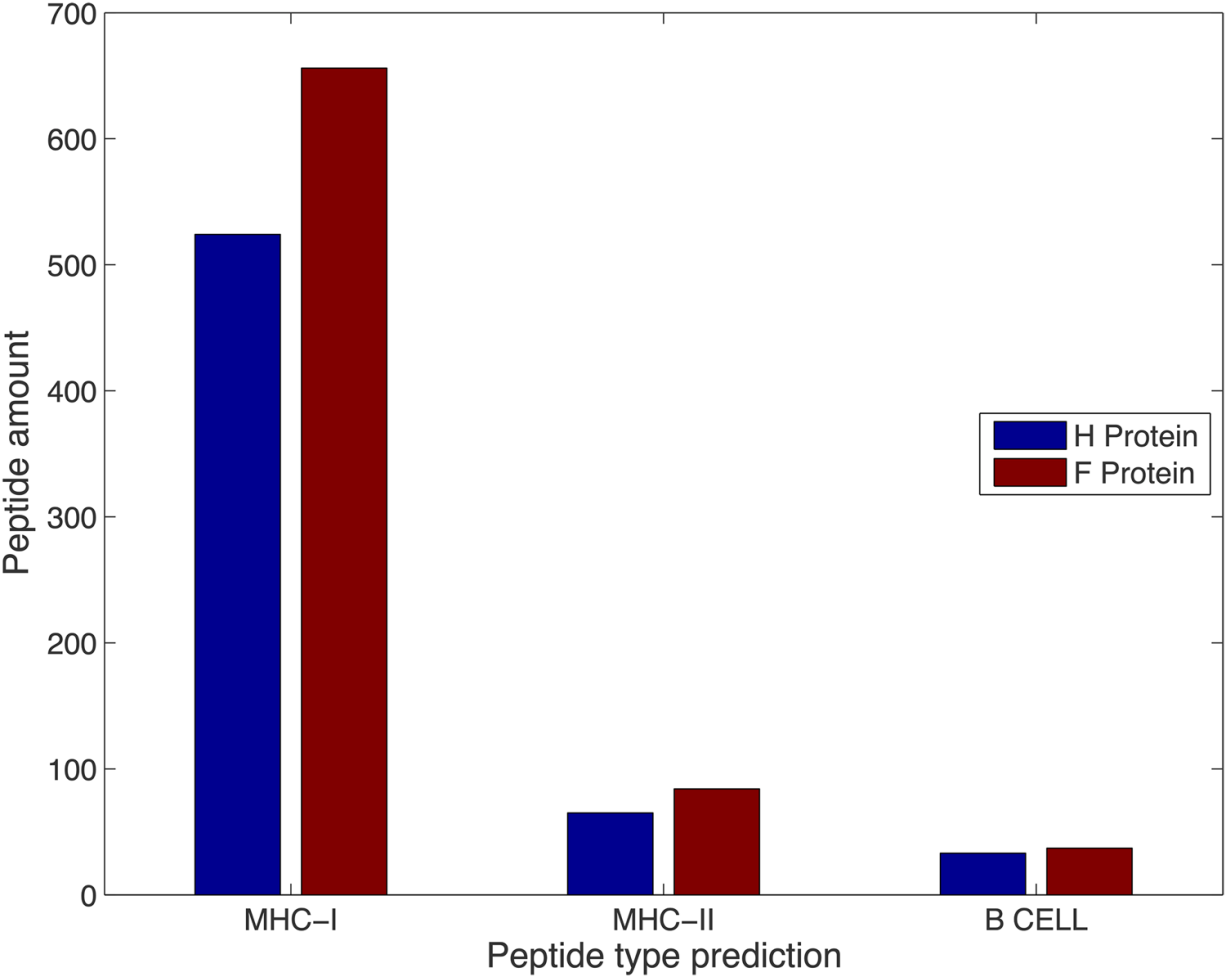
Total predicted peptides from H and F CDV proteins for CD8+ T cytotoxic cells (MHC-I), helper CD4+ T cells (MHC-II), and linear B cells. Computational tools such as MHC2PRED, CTLPRED, IEDB from the La Jolla Institute, and SVMTRIP were used to predict the immunogenic peptides based on the consensus sequence of the H and F CDV proteins.

After a consensus sequence of H and F CDV proteins from all reported linages was assembled, we selected peptides that were immunogenic to B cells linearly and those predicted to induce CD4+ and CD8+ T-cell responses, all of which could be employed as vaccine formulations. A total of 1399 peptides were predicted based on the best score from the consensus sequence of the H and F CDV proteins (Fig. 1). The helper CD4+ T-cell epitopes were more abundant for both the H and F proteins, and the linear B-cell epitopes were less abundant for both CDV proteins. Thus, a peptide library with immunogenic potential was constructed to determine the most promising antigens in a peptide-based vaccine.

### Candidate peptide selection and physicochemical properties

From the total amount of predicted peptides, 12 peptides were selected based on the highest prediction score issued by the prediction tools and their physicochemical properties, such as charge, length, stability, and predicted half-life (Table 1). All peptides are classified as epitopes for CD8+ T cytotoxic cells (PCIs), helper CD4+ T cells (PCIIs), and linear B cells (LIBBs) from either the H or F protein. Importantly, after computational prediction, the peptides PCII-F3-012 and PCI-F3-038 had the same sequence and were predicted to have immunogenic potential for both CD8+ T cytotoxic cells and helper CD4+ T cells. Regarding the physicochemical properties, as shown in Table 1, the charges of the selected peptides are between 0 and + 2, except LINB-IEDB-H, which has a charge of − 1; most of them have a favorable instability index, indicating their stable nature, and the length of the peptides oscillates between 9 and 13 amino acids. The half-life in hours varies between 1 and 100 h, which is important for the use of a potential vaccine candidate. Therefore, we concluded that the peptides selected based on the best score exhibited favorable physicochemical properties and could be considered vaccine candidates.

**Table 1:** Selected peptides and their physicochemical properties.

ID	Peptide	Length (amino acids)	Position	Charge	Molecular weight (g/mol)	Stability	Half-life (h)
P1	PCI-H1-004	9	36	0	1090.41	+	> 20
P2	PCI-H1-030	11	97	0	1272.55	+	1
P3	PCII-H1-008	9	592	1	1145.37	+	100
P4	PCI-F3-053	12	651	2	1311.50	+	5.5
P5	PCII-F3-012	9	316	1	1198.43	+	5.5
P6	PCI-IEDB-H1	9	473	0	1026.24	+	7.2
P7	LINB-IEDB-H	11	367	− 1	1321.42	+/−	100
P8	PCI-F3-054	12	645	1	1261.44	+	> 20
P9	PCII-IEDB-H2	13	32	0	1512.98	+	> 20
P10	PCI-IEDB-F1	9	552	0	1039.26	+	30
P11	PCII-IEDB-F2	13	488	2	1360.62	+/−	30
P12	LINB-IEDB-F	9	134	1	1123.28	+	1.3

### Homology modeling, molecular docking, and molecular dynamics

**Figure 2:**
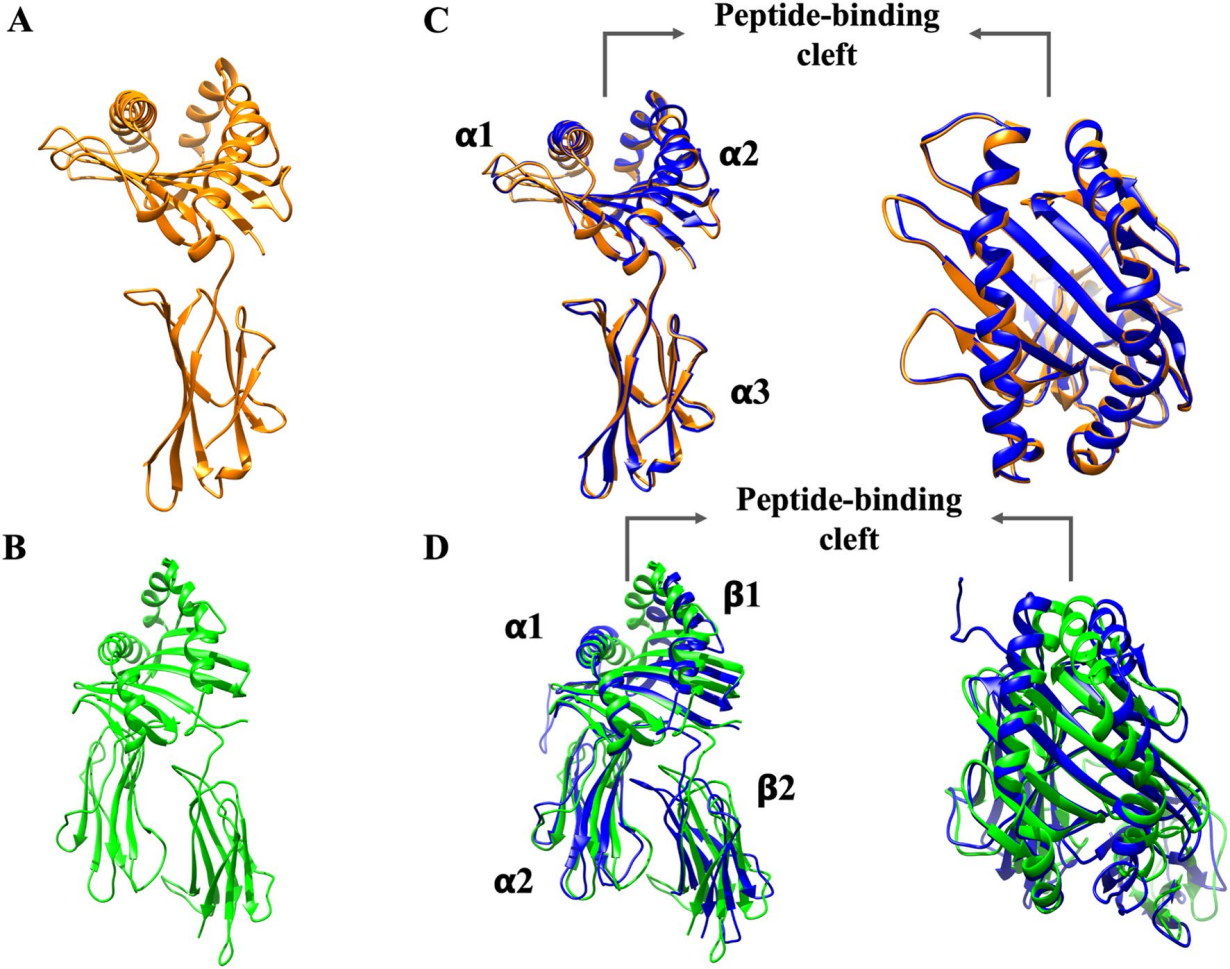
Canine MHC molecule and TLR homology models obtained by MODELLER v. 10.0 and structural alignments with PDB template structures. (**A**) Canine MHC class I molecule homology model (orange). (**B**) Canine MHC class II molecule homology model (green). (**C**) Structural alignment of the template (PDB code: 5F1N) (blue) and canine MHC class I molecule homology model (orange). (**D**) Structural alignment of a template (PDB code: 4FQX) (blue) and canine MHC class I molecule homology model (green). Homology modeling was performed using MODELLER v. 10.0. The peptide-binding cleft was marked in both MHC molecules.

Due to the lack of crystallographic structures from either canine MHC molecules or TLRs, homology modeling was employed to determine the 3D structure, which could allow the interaction of selected canine MHC molecule peptides with TLRs to be assayed. A 3D model of canine MHC molecules and TLRs is shown in Fig. 2A,B. These models were carefully validated through computational tools. Ramachandran plots revealed that more than 95% of the amino acids in all the models were located in favorable regions for rotation and torsion (data not shown). The overall model quality calculated with ProSA-Web indicated that all models are located within the distribution of all the proteins in the PDB that come from X-ray crystallography (Supplementary Table 1). The TM values indicated that all the models had global folding identical to that of the template, with values close to one (Supplementary Table 1). Structural alignment with the templates was carried out (Fig. 2C,D). Both the models and the templated model exhibit overall folding since they can match after alignment. To observe the capacity of immunogenic peptides to interact with either canine MCH molecules or TLRs, molecular docking was employed.

Three different tools were used, and the molecular docking scores of the selected peptides are reported in Table 2. The peptide PCI-F3-053 exhibited a favorable docking score in all the tools used, indicating a consensus prediction. Moreover, the PCII-IEDB-H2 peptide obtained the best docking score only with MDOCKPEP, and the PCI-H1-004 peptide had the best docking score with the HPEPDOCK server. Peptide PCI-F3-054 exhibited a low cluster density but the highest number of cluster elements, indicating that the 3D structure in this cluster is the most likely among the predicted poses, according to CABSDOCK. In contrast, the peptide PCI-F3-053 exhibited the highest cluster density because of the low number of elements, indicating a less probable pose among the predicted poses.

**Table 2:** Molecular docking scores obtained with different computational tools for selected peptides with canine MHC molecules.

ID	Peptide	HPEPDOCK	MDOCKPEP	CABSDOCK		
				Cluster density	RMSD	Elements
P1	PCI-H1-004	− 308.716	− 184.3	75.4363	1.829	138
P2	PCI-H1-030	− 221.010	− 189.3	56.5631	2.333	132
P3	PCII-H1-008	− 214.753	− 167.3	86.7120	1.614	140
P4	PCI-F3-053	− 224.458	− 215.3	156.2350	0.294	46
P5	PCII-F3-012	− 220.671	− 189.3	105.4340	0.882	93
P5.1	PCI-F3-038*	− 252.723	− 200.4	98.3842	1.128	111
P6	PCI-IEDB-H1	− 249.353	− 173.9	101.203	1.324	134
P7	LINB-IEDB-H**	NA	NA	NA	NA	NA
P8	PCI-F3-054	− 251.275	− 173.2	54.0866	4.918	266
P9	PCII-IEDB-H2	− 225.170	− 218.0	96.4651	1.057	102
P10	PCI-IEDB-F1	− 281.525	− 162.1	46.7683	3.057	143
P11	PCII-IEDB-F2	− 228.006	− 186.2	54.9054	1.821	100
P12	LINB-IEDB-F**	NA	NA	NA	NA	NA

*This peptide has the same sequence as P5; however, it was predicted to be present in both MHC class I and II.

**There is no docking score for linear B-cell epitopes interacting with MHC molecules.

For cTLR-2 and cTLR-4, the HPEPDOCK server was used to assay the interaction of the selected peptides with the innate immune receptors. The peptide PCI-H1-004 had the greatest interaction with TLR-2, and LINB-IEDB-F had the greatest interaction with TLR-4 (Table 3). Therefore, we conclude that all peptides obtained favorable docking scores when employing different computational tools, considering that all docking servers were blind, which means that none of them had a previous location to interact and that all peptides were in the peptide-binding cleft, which is the most likely interaction area.

**Table 3:** Molecular docking scores obtained with different computational tools for selected peptides containing canine TLR-2 and TLR-4.

ID	Peptide	TLR-2	TLR-4
P1	PCI-H1-004	− 250.001	− 177.975
P2	PCI-H1-030	− 182.783	− 183.632
P3	PCII-H1-008	192.197	− 176.631
P4	PCI-F3-053	− 215.147	− 185.100
P5	PCII-F3-012	− 211.496	− 180.756
P5.1	PCI-F3-038*		
P6	PCI-IEDB-H1	− 219.952	− 178.147
P7	LINB-IEDB-H	− 143.050	− 138.441
P8	PCI-F3-054	− 210.443	− 164.827
P9	PCII-IEDB-H2	− 207.528	− 177.352
P10	PCI-IEDB-F1	− 213.697	− 155.800
P11	PCII-IEDB-F2	− 191.366	− 163.752
P12	LINB-IEDB-F	− 209.526	− 194.692

*This peptide has the same sequence as P5; however, it was predicted to be present in both MHC class I and II.

**Figure 3:**
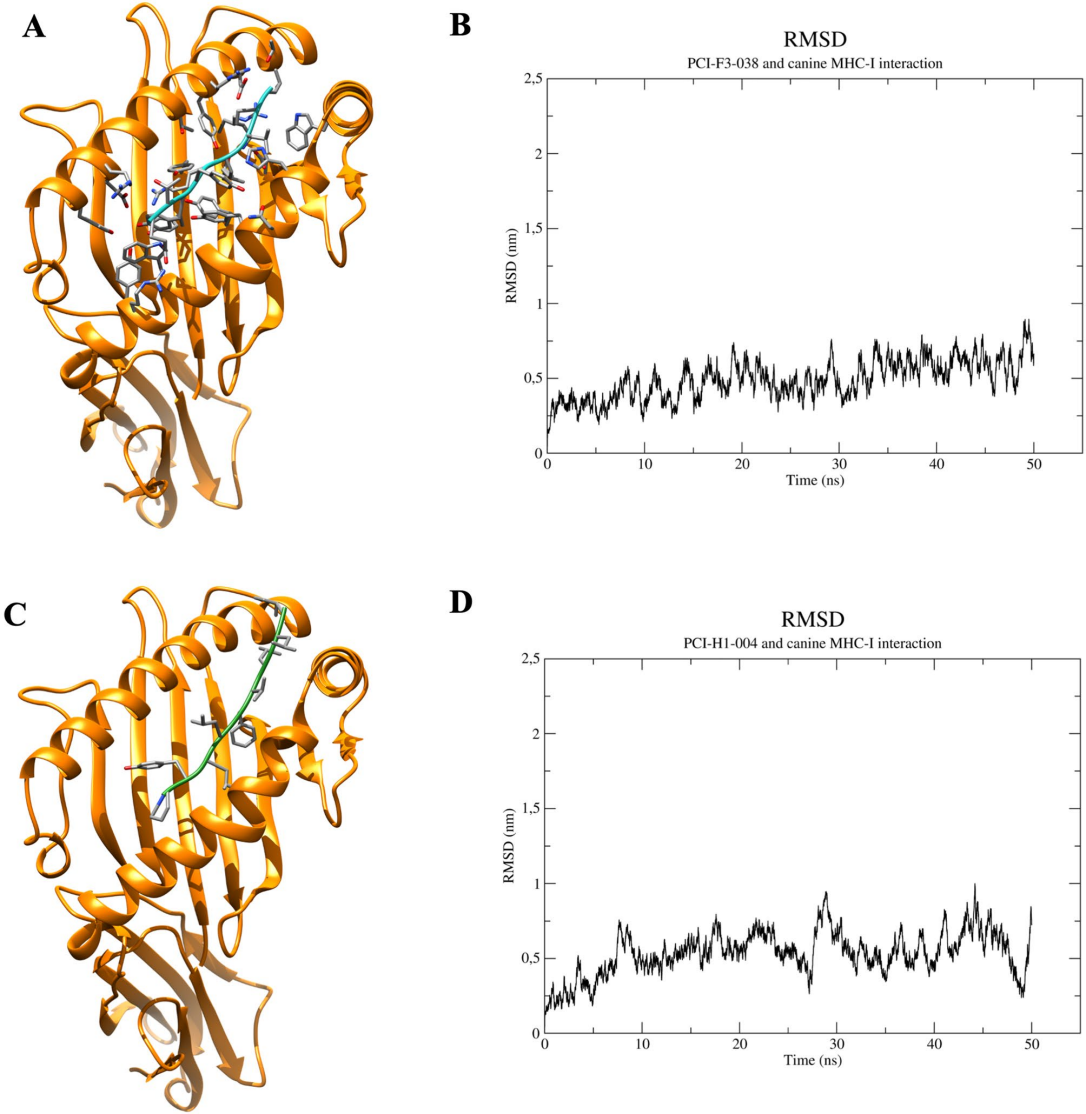
Molecular docking and dynamics of the predicted peptides in complex with MHC molecules. (**A**) Molecular interaction between PCI-F3-038 and canine MHC-I molecules obtained with a molecular docking tool. (**B**) Root mean square deviation (RMSD) plot of the interaction interface between PCI-F3-053 and MHC class I. (**C**) Molecular interaction between PCI-H1-004 and canine MHC-I molecules obtained with a molecular docking tool. (**D**) RMSD deviation plot of the PCI-F3-004 and MHC class I interactions. Representative peptide–MHC complex molecules are shown. All 3D graphics were generated using the software UCSF Chimera. Graphs for molecular dynamics were obtained with Xmgrace software (Oregon Graduate Institute of Science and Technology, Hillsboro, OR, USA).

Molecular docking complexes from PCI-F3-038 and canine MHC-I (Fig. 3A) were employed as the starting structure for molecular dynamic simulations to determine whether interactions between the selected peptides and MHC and TLRs are stable over time. As shown in Fig. 3B, the peptide PCI-F3-038 tended to be stable after 30 ns of simulation, with an RMSD between 0.5 and 0.8 nm. On the other hand, the complex from PCI-0H1-004 and MHC-I (Fig. 3C) exhibited a nonstable trend, oscillating from 0 to 1 after 50 ns of simulation, indicating that the peptide from PCI-0H1-004 and the MHC-I complex could be less stable than that from PCI-F3-038 (Fig. 3D). Notably, molecular docking complexes were obtained with blind-docking tools since both structures were submitted separately; however, in the complexes shown in Fig. 3A,C, both peptides were located in the peptide-binding cleft, allowing us to wonder about the capacity of those peptides to interact with MHC-I because the molecular docking tool indicates the most likely pose after hundreds of evaluated poses. Other molecular dynamics simulations between selected peptides in complex with canine MHC molecules are shown in Supplementary Fig. 2.

### In silico safety evaluation through computational tools

To determine the potential use of the selected peptides, we conducted an in silico safety evaluation through multiple computational tools to assess antigenicity, allergenicity, and toxicity. As shown in Table 4, some peptides have the potential to be allergens (PCI-H1-004, PCI-F3-053, PCI-IEDB-H1, PCI-F3-054, PCII-IEDB-H2, and LINB-IEDB-F). Furthermore, all selected peptides were determined to be nontoxic to ToxinPred, and when antigenicity was evaluated, most peptides exhibited antigenic potential, except for PCI-H1-030, PCI-H1-030, and PCII-IEDB-F2, which had negative values (Table 4). Additionally, some of them were not classified as antigens; however, they were predicted to be immunogenic peptides with other computational tools (Fig. 1). In addition, the sequence homology between the peptides and canine proteome was determined, revealing that there were no peptides with 100% coverage and that the identity was any match, indicating that no identical peptide was found in the canine proteome. BLAST homology assessment indicated that the predicted peptide vaccine did not cause autoimmune responses to the host since no peptide was identical in any canine protein (Supplementary Table 2). In summary, all peptides have been demonstrated to be nontoxic, and some of them have the potential to be allergenic since they have a potential prediction score.

**Table 4:** In silico safety assessment of the antigenicity, allergenicity, and toxicity of selected peptides.

ID	Peptide	ToxinPred^a^	AllergenFP^b^	VaxiJen^c^
		Toxic/non-toxic	Allergen/non-allergen	Antigen/non-antigen
P1	PCI-H1-004	Non-toxic	Allergen	Antigen
P2	PCI-H1-030	Non-toxic	Non-allergen	Non-antigen
P3	PCII-H1-008	Non-toxic	Non-allergen	Antigen
P4	PCI-F3-053	Non-toxic	Allergen	Antigen
P5*	PCII-F3-012	Non-toxic	Non-allergen	Non-antigen
P6	PCI-IEDB-H1	Non-toxic	Allergen	Antigen
P7	LINB-IEDB-H	Non-toxic	Non-allergen	Antigen
P8	PCI-F3-054	Non-toxic	Allergen	Antigen
P9	PCII-IEDB-H2	Non-toxic	Allergen	Antigen
P10	PCI-IEDB-F1	Non-toxic	Non-allergen	Antigen
P11	PCII-IEDB-F2	Non-toxic	Non-allergen	Non-antigen
P12	LINB-IEDB-F	Non-toxic	Allergen	Antigen

*This peptide has the same sequence as P5; however, it was predicted to be present in both MHC class I and II.

^a^ToxinPred determines whether peptides are toxic in the 10 amino acid window, with a dichotomic result indicating toxicity or nontoxicity.

^b^AllergenFP predicts the probability of allergenicity with a dichotomic result as probable allergen or probable no allergen.

^c^VaxiJen prediction of antigens and subunit vaccines with a dichotomous result as a viral antigen or nonviral antigen.

### In vitro safety evaluation of selected peptides in cell lines demonstrated their low cytotoxicity

**Figure 4:**
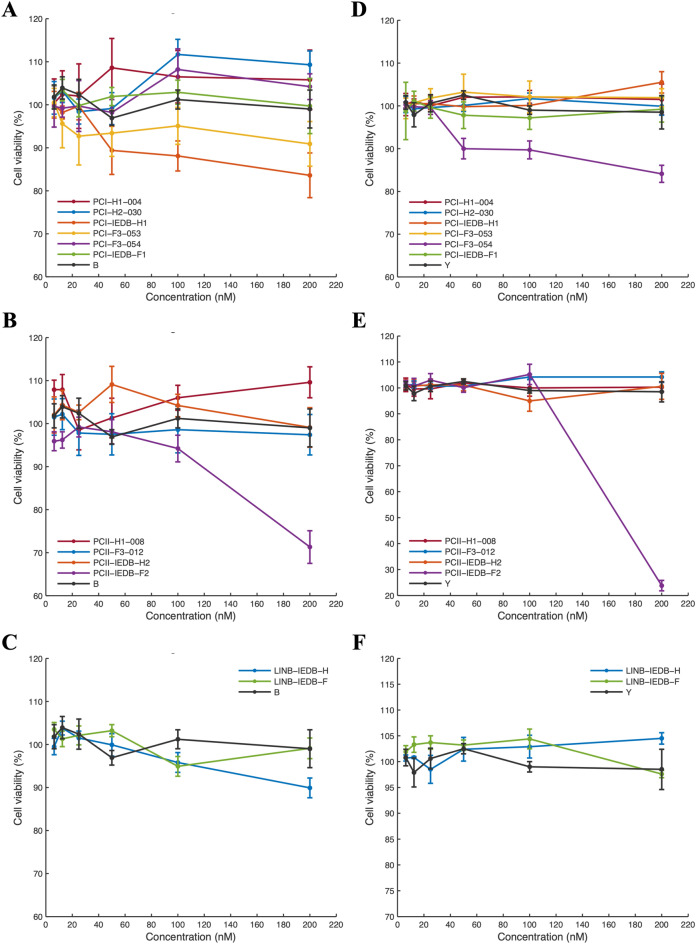
MTT assay for in vitro evaluation of the viability of selected peptide-treated Vero-Dog-SLAM and MDCK cells. (**A**) Cell viability was evaluated in the Vero-Dog-SLAM cell line after treatment with the predicted MHC class I peptides. (**B**) Cell viability was evaluated in the Vero-Dog-SLAM cell line after treatment with the predicted MHC class II peptides. (**C**) Cell viability was evaluated in the Vero-Dog-SLAM cell line after treatment with linear B-cell-predicted peptides. (**D**) Cell viability was evaluated in the MDCK cell line after treatment with the predicted MHC class I peptide. (**E**) Cell viability was evaluated in the MDCK cell line after treatment with the predicted MHC class II peptide. (**F**) Cell viability was evaluated in the MDCK cell line after treatment with linear B-cell-predicted peptides. Cells were treated with twofold serial dilutions of selected peptides ranging from 6.125 to 200 nM for 48 h. Cells without peptide were used as a viability negative control, and cells treated with 0.5% Triton X-100® served as a cytotoxicity positive control. Two independent experiments with 4 replicates (n = 8) were carried out for each cell line, and nontreated cells were used as negative controls. Peptides B and Y, which have reported antimicrobial activity, were used as noncytotoxic control peptides. The means and coefficients of variation are shown in the graph.

The in vitro safety of the selected peptides was determined after chemical synthesis in cell lines. To establish whether the peptides were cytotoxic, different cell lines were treated with several dilutions of peptides. For Vero-Dog-SLAM, most peptides exhibited cell viability greater than 90% at low concentrations (< 50 nM) (Fig. 4A–C). Thus, the peptides PCI-IEDB-H1 and PCII-IEDB-F2 showed cell viability lower than 90% at concentrations higher than 100 nM. For MDCK cells, similar to Vero-Dog-SLAM cells, most peptides exhibited cell viability greater than 90% at low concentrations (< 50 nM) in MDCK cells. However, the viability of cells treated with the peptides PCI-F3-054 and PCII-IEDB-F2 was lower than 90% at concentrations higher than 100 nM (Fig. 4D–F). Both the Y and B control peptides had a cell viability of approximately 100% at all the evaluated concentrations (Fig. 4). We concluded that most peptides were noncytotoxic at the evaluated concentrations when treated with two cell lines, one from a dog kidney and another expressing the dog SLAM receptor, which are important for virus recognition and entry into the cell, except for the peptides PCI-IEDB-H1, PCII-IEDB-F2 and PCI-F3-054.

### The selected peptides were safe for use in cPBMCs and cRBCs

**Figure 5:**
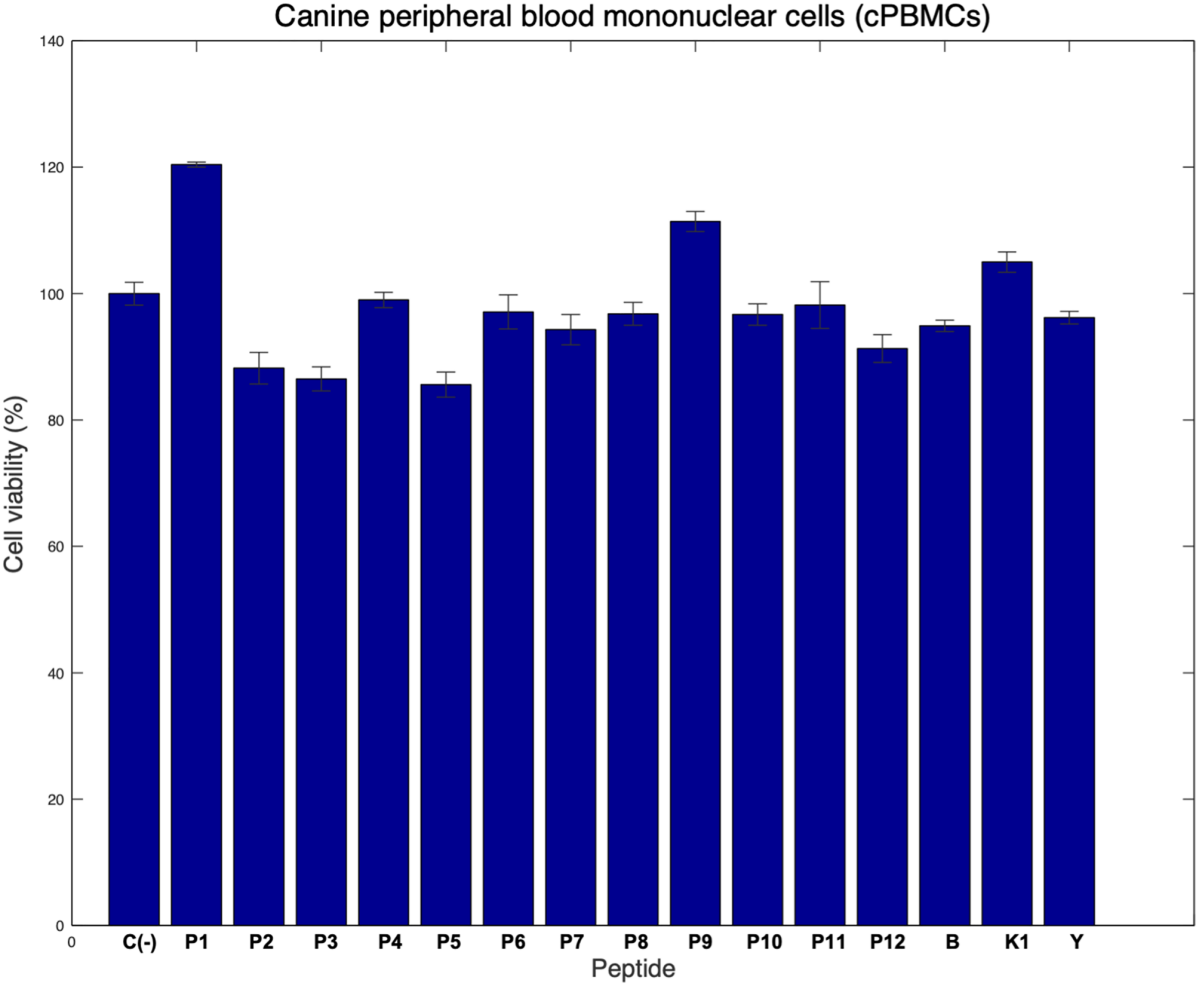
In vitro selected peptide safety evaluation in canine primary cells. Evaluation of cell viability after treatment with selected peptides in cPBMCs through the MTT assay. Cells were treated with the selected peptides at 100 nM for 48 h. Cells without peptide were used as a viability negative control, and cells treated with 0.5% Triton X-100® served as a cytotoxicity positive control. Two independent experiments with 4 replicates (n = 8) were carried out, and nontreated cells were used as negative controls. Peptides B, K1, and Y, which have reported antimicrobial activity, were used as noncytotoxic control peptides. The means and coefficients of variation are shown in the bar graph.

To confirm the results obtained in the cell lines, we performed an assay in primary cells from dogs to determine whether the selected peptides were cytotoxic when exposing cRBCs and cPBMCs to them. When cytotoxicity was evaluated in cRBCs, most peptides had a hemolysis percentage lower than 2% at all assayed concentrations, except for the peptide PCII-IEDB-F2 (Table 5). This peptide exhibited allowed hemolysis of less than 2% at concentrations less than or equal to 25 nM. Therefore, we concluded that most peptides exhibited a low potential cytotoxic effect on primary cells, cPBMCs, and cRBCs, except for the peptide PCII-IEDB-F2, which has high hemolytic potential. As shown in Fig. 5, all the evaluated peptides, including the control peptides B, K1, and Y, exhibited cell viability greater than 80% in the cPBMCs. Most of the peptides had cell viability close to 100% compared to that of untreated cells. However, PCI-H1-030, PCII-H1-008, and PCII-F3-012 resulted in approximately 80% cell viability. On the other hand, the viability of the PCI-H1-004-treated cells was greater than that of the nontreated cells (Fig. 5).

**Table 5:** Hemolytic potential of selected peptides in canine RBCs.

ID	Peptide	Safe concentration (nM)	Hemolytic (yes/no)
P1	PCI-H1-004	< 200	No
P2	PCI-H1-030	< 200	No
P3	PCII-H1-008	< 200	No
P4	PCI-F3-053	< 200	No
P5	PCII-F3-012	< 200	No
P6	PCI-IEDB-H1	< 200	No
P7	LINB-IEDB-H	< 200	No
P8	PCI-F3-054	< 200	No
P9	PCII-IEDB-H2	< 200	No
P10	PCI-IEDB-F1	< 200	No
P11	PCII-IEDB-F2	< 25	No
P12	LINB-IEDB-F	< 200	No

PBS was used as a negative hemolytic control, and 0.1% Triton X-100® was used as a positive hemolytic control. Two independent experiments with 4 replicates (n = 8) were carried out. The percent hemolysis was calculated as the relationship between the treatment and control absorbance, and a safe concentration was reported.

### Multiepitope polypeptide exhibited a safe profile in silico and in vitro

**Figure 6:**
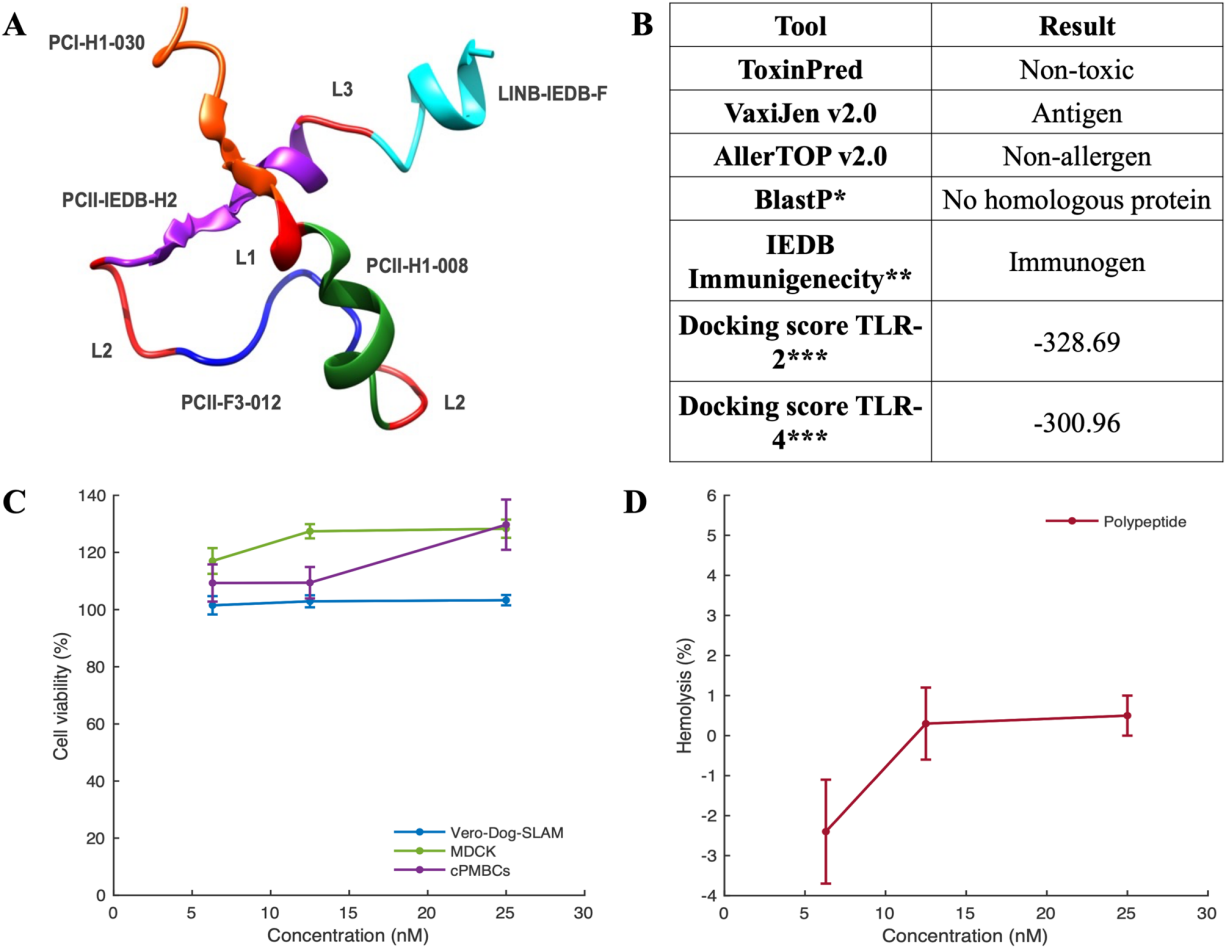
Multiepitope polypeptide characteristics and in silico and in vitro safety validation. (**A**) Structural model of the multiepitope polypeptide obtained with I-TASSER. (**B**) In silico safety validation data for the polypeptide. (**C**) Evaluation of the viability of Vero-Dog-SLAM, MDCK and cPBMC cells after polypeptide treatment through the MTT assay. Cells were treated with twofold serial dilutions of polypeptide from 6.125 to 25 nM for 48 h. Cells without polypeptide were used as a viability negative control, and cells treated with 0.5% Triton X-100® served as a cytotoxicity positive control. (**D**) Hemolytic potential of the polypeptide in canine RBCs. PBS was used as a negative hemolytic control, and 0.1% Triton X-100® was used as a positive hemolytic control. Two independent experiments with 4 replicates (n = 8) were carried out, and nontreated cells were used as negative controls. The means and coefficients of variation are shown in the graph. *Evaluated with the *Canis lupus familiaris* proteome as the dataset. **Score of 0.57349, classified as a probable antigen MCH-I. ***Docking score obtained with the HPEPDOCK tool.

After peptide validation, a polypeptide composed of PCI-H1-030, PCII-H1-008, PCII-F3-012, PCII-IEDB-H2 and LINB-IEDB-F was constructed based on the best peptides that overcome all in silico and in vitro safety assays. L1, L2 and L3 refer to amino acid linkers such as AYY, GPGPG and KK, respectively. A threading model with I-TASSER was obtained to determine the 3-D structure (Fig. 6A). Then, we performed an in silico safety evaluation through multiple computational tools to assess antigenicity, allergenicity, and toxicity. Polypeptide was demonstrated to be safe in silico since all the employed validations exhibited acceptable values (Fig. 6B). Notably, the polypeptide has an IEDB score of 0.57349, which is classified as a probable antigen from MCH-I. Additionally, no homologous proteins in the canine proteome were found, indicating that there is no risk of developing autoimmunity (Fig. 6B). The in vitro safety of the peptides was assessed similarly to that of single peptides in cellular models such as Vero-Dog-SLAM, MDCK and cPBMCs to determine whether the polypeptide was cytotoxic when it was treated with several dilutions. As shown in Fig. 6C, the polypeptide exhibited a cell viability greater than 90% at low concentrations (< 25 nM) in both cell lines and primary cells as cPBMCs. On the other hand, when cytotoxicity was evaluated in cRBCs, polypeptides had a hemolysis percentage lower than 2% at all assayed concentrations, from 6.125 to 25 nM, similar to that of selected single peptides (Fig. 6D).

## Discussion

Vaccine development has played an important role in human and animal health. For CDV, there are either conventional or recombinant vaccines based on the Onderstepoort strain^3^. However, neither peptides nor inactivated vaccines are available. New-generation vaccines have been employed as important development platforms since they have been used in animals and humans^28,29^. In dogs and humans, recombinant vaccines have been used, and there are different recombinant vaccines for CDV^13^, Ebola^30^, Dengue^31^, and SARS-CoV-2^32^. Diverse computational tools have been employed for vaccine design because multiple candidates can be identified reducing not only in vitro experiments but also, the risk of pathogens propagation in wet laboratory^33^. Vaccine alternatives for diverse viral agents include vaccinomics, which may contribute to the design of vaccine candidates^34^. Although CDV is not considered a threat to humans, in animals, it is a highly contagious disease that can affect a wide range of domestic and wild animals, including some that are endangered^35,36^. Therefore, an effective vaccine candidate must be designed since the emergence of new variants has made CDV a threat to animal health and well-being since there is a high mortality rate among infected animals^37^.

We generated a peptide library based on the main antigenic determinants of CDV, proteins H and F, employing a consensus sequence that considered all reported sequences from the CDV lineage via diverse computational tools (Fig. 1). These tools are assembled with machine learning, indicating their immunogenic potential based on known peptides employed in machine training. Selection based on the best score and physicochemical data, including antigenicity, allergenicity, and toxicity profile, was subsequently performed, and the results were submitted to in silico safety validation (Table 1), since vaccines can stimulate the immune system and cause allergic reactions. A protein sequence is considered potentially allergenic if its sequence has at least six contiguous amino acid identities within the range of 80 amino acids, a sequence identity of 0.35%, with a known allergen^38^. Therefore, the most promising antigenic peptides identified in this study have nonallergenic potential.

To induce a prolonged and effective immune response, both B-cell and T-cell functions are required to promote not only humoral but also cellular immunity mediated by the cells mentioned above^27^. Here, we predicted helper CD4+ T cells, CD8+ T cytotoxic cells, and linear B-cell epitopes to obtain a great number of prospective epitopes that will have the ability to prompt a robust immune response since the B-cell epitope of a target molecule must combine with a T-cell epitope; thus, a peptide vaccine can be considerably immunogenic^27^. There is an essential step in immune response development for epitopes, which consists of the recognition of epitopes for MHC molecules^27^. In dogs, MHC alleles have diverse characteristics regarding variability, which could imply consequences for peptide immunogenic effects and presentation. The class I region contains one highly polymorphic gene, DLA-88, in addition to several other genes^39^. The class II molecular region includes four genes, DLA-DRA1, which seems to be monomorphic, and DLA-DRB1, DQA1, and DQB1, which have been reported to be highly polymorphic^39^. Currently, 106 DLA-DRB1, 26 DLA-DQA1, and 62 DLA-DQB1 alleles have been identified in dogs and other related canids^40^. However, there is a lack of within-breed variability in MHC alleles expressed in dogs, and it has been reported that the English springer spaniel exhibits a slightly above-average diversity of MHC alleles^41^. Nevertheless, diverse peptides that can represent not only all CDV lineages but also the capacity to be presented in any MHC molecule context must be generated to develop new-generation vaccines that increase immunization capacity.

Homology modeling is a helpful tool for elucidating the 3D structure and interactions of proteins because the number of reported sequences in databases is greater than the number of crystallographic structures in the PDB^42,43^. This is the case for canine MHC molecules and TLRs since no crystallographic structures are available due to a lack of experimental data. We used homology modeling to model canine MHC molecules and TLRs based on human MHC molecules and TLRs reported in the PDB (Fig. 2, Supplementary Table 1). Moreover, molecular docking was employed to determine whether selected peptides could interact with modeled canine MHC molecules (Table 2, Fig. 2) and TLRs (Table 3) to predict their molecular interaction, since several in silico approaches have demonstrated potential vaccine candidates through techniques such as molecular docking and dynamics, employing other viral models as targets, such as Dengue, Canine Circovirus and Marburg virus^17,44,45^. The docking study resulted in negative values of binding energy (Tables 2, 3), which demonstrates the potential high binding affinity between peptides and canine MHC molecules and between TLR-2 and TLR-4 since the H protein from another Morbillivirus virus, such as the measles virus, has been shown to interact with these PRRs^46,47^. Thus, these interactions with TLR-4 may elicit a protective innate immune response. Notably, peptides that can interact not only with MHC molecules but also with PRRs may have a coadjuvant function since they are dedicated to identifying PAMPS from diverse microorganisms. After TLR recognition and activation by viral ligands, cytokine production, in addition to the upregulation of MHC molecules, issues the link between the adaptive immune response and the innate immune system^48^. Considering the low immunogenicity of peptides in comparison to live-modified attenuated vaccines, their coadjutant function could be an important advantage of peptide-based vaccines^49^.

Molecular docking and dynamics enable us to demonstrate at least computationally the possibility of selected peptides interacting with MHC molecules (Fig. 3, Supplementary Fig. 2) since blind-docking tools were employed to investigate the capacity of selected peptides to interact with MHC molecules. As expected, selected peptides that were predicted to have immunogenic potential could interact spontaneously in the peptide-binding cleft (Fig. 3A,C), and some interactions must also be stable over time (Fig. 3B, Supplementary Fig. 2). Several studies have shown similar approaches to determine whether potential peptides or polypeptides could interact with molecules from the host immune system, indicating, at least from a computational approach, potential protein-based new vaccines for human and veterinary viral agents^38,44,45^.

Peptide properties and physicochemical characteristics are major concerns in active peptide development, as the half-life of peptides is considered a challenging limitation^50,51^. In this work, there was a wide range of values for the half-life measured by the ProtParam tool, which led to the consideration of the stability of immunogens in a potential peptide-based vaccine (Table 1). Although some peptides have a low half-life, their capacity to interact with TLRs (Table 3) indicates their coadjuvant properties in combination with an adjuvant in a future new peptide-based vaccine, as has been proposed for other viral agents^23,44^. Additionally, the stability of all the selected peptides was measured, and the results indicated that most of the peptides are stable in biological environments (Table 1). The half-life, stability and even immunogenicity of peptides could be easily improved by the introduction of nonnatural amino acids and peptide-like molecules into peptides, enabling the development of vaccines based on rational drug design^27^. Peptide charge plays an important role considering that those abundant in amino acids with positive charges attaches to the negatively charged lipid bilayer of red blood cells. This attachment causes the membrane to break down, enabling water and other molecules to penetrate the cell. Consequently, the osmotic pressure within the red blood cell rises, resulting in cell enlargement and eventual rupture^52,53^. Therefore, a peptides a PCII-IEDB-F2 must be improved through charge decreasing (Table 1). On the other hand, homology peptides were searched in the dog proteome, there was no identical peptide in canine proteins (Supplementary Table 2), reducing the risk of autoimmune diseases after immunization with selected peptides^17^. Thus, to guarantee some aspects, such as charge, homology with host proteins, stability, and half-life, reported in other studies to demonstrate the potential of immunogenic peptides^17^, our predicted peptides exhibited potential activity, at least from a computational perspective.

After the in silico approach, in vitro studies were performed to evaluate the characteristics, such as the cytotoxicity and hemolytic potential, of the selected peptides to elucidate whether they could be used as alternative vaccines composed of multiple peptides oriented toward stimulating the main aspects of the immune response. Here, we evaluated the cytotoxicity of selected peptides in Vero-Dog-SLAM and MDCK cells (Fig. 4) and in primary PBMCs (Fig. 5), indicating, from an in vitro perspective, the potential safety of the selected peptides. Moreover, the hemolytic potential was assayed (Table 5), and most peptides exhibited a hemolytic percentage lower than 2%, indicating their nonhemolytic profile. These results lead to questions about the safety of the selected peptides since these kinds of molecules have been reported to be highly safe^27^ because this material lacks infectious material that can support live or attenuated vaccines, and there is no risk of reversion that can lead to virulence, which is a potential issue with these kinds of vaccines. There is no risk of genetic integration or recombination, which is a problem facing regulatory authorities that are dealing with DNA vaccines^27^. However, DNA vaccines could be more cost-effective than other vaccines, such as peptide vaccines, and they are also considered next-generation vaccines^54^, in addition to some disadvantages that peptide vaccines must overcome, such as reduced immunogenicity compared to that of live attenuated vaccines^55^, the limited range of immune recognition and potential ineffectiveness in individuals with diverse MHC profiles^27^, the need for the incorporation of multiple epitopes to elicit robust immune responses^56^, and the need for the induction of short-lived immune responses^57^. However, some interesting characteristics of immunogenic peptides include the insertion of other chemical groups, such as lipids, carbohydrates, and phosphates, to improve their immunogenicity, stability, and solubility. Moreover, peptide formulations can be stored easily, which avoids the need for low-temperature storage and facilitates transport and distribution^27^. Considering that peptide vaccine development has emerged as an interesting alternative for other viruses including CD4+ and CD8+ T-cell epitopes^58,59^, the induction of cellular immunity may have broad advantages in defense against CDV, since cross-cellular immunity seems to offer grater chances for obtaining protection^60,61^, based on CDV biology^3^. Finally, the inclusion of multiple immunogenic peptides, as single peptides in a mixture or as multiepitope immunogen polypeptides, is an important topic of wide discussion^27^. Several studies have elucidated the importance and usage of vaccinomics, the employment of “omics”, in the field of vaccinology to develop vaccine candidates^18,19^. New candidate peptides-based vaccines have been developed with two alternatives: multipeptide vaccines, comprising different single peptide or multiepitope polypeptide vaccines, combined with peptide linkers with potential coadjuvant activity^62^.

In TCR–MHC structures, the TCR contacts both the peptide antigen and the MHC. The peptide, despite its small size compared to that of the MHC molecule, can contribute greatly to the buried surface area or peptide binding cleft of the MHC, indicating the contribution of potential immunogenic peptide interactions with MHC molecules in rational epitope discovery to the design of next-generation vaccines based on peptides^63^. A significant positive correlation between the TCR affinity and the TCR–MHC binding cleft has been found, even though it could be expected that an increase in the number of molecular interactions and contacts, no matter their power, must potentially contribute and accumulate toward a higher affinity^63^. This is a crucial parameter for T-cell activation, and there is a direct link between the structural parameters of the TCR–MHC complexes that impact T-cell function, in contrast to the importance of peptides that interact in the MHC binding cleft^64,65^. For CTLs, the employed method allows the prediction of epitopes using quantitative matrix and machine learning techniques such as support vector machine and artificial neural network approaches restricted to MHC-I in predicted T-cell epitopes. Subgroup analysis can discriminate between T-cell epitopes and other MHC binders and nonepitopes^66^. The SMM-align method employed for MHC-II prediction methods enables the determination of quantitative peptide-MHC-II binding affinity values, which makes this tool suitable for rational epitope discovery since the prediction method was trained and evaluated on a publicly available dataset for nine HLA-DR supertypes^66^. For linear B-cell epitopes, we used a computational tool capable of distinguishing virus peptides, and hence, this tool has a greater chance to correctly predict with a sensitivity higher than 80%, indicating a considerable probability of reaching potential linear B-cell epitopes^67^. Therefore, all the computational tools employed for the prediction of CDV immunogenic peptides, as well as those reported in the literature, are supported by robust computational and experimental methods that reliably predict potential immunogenic peptides.

To recover immunogenic peptides, defective ribosomal initiation products must be analyzed. Once MHC class I molecules are loaded with immunogenic peptides, they may be protected from proteolysis, and these complexes pass through the Golgi apparatus following their way to the cell surface, where they are sensed by CD8+ T cells^68^. Furthermore, when referring to diverse mechanisms in the context of MHC class II molecules, the process could involve intact exogenous antigens since both must be loaded into the MHC class II molecules in the antigen-binding cleft. As MHC class I-targeted epitopes, immunogenic peptides can reach MHC class II molecules and replace existing peptides via a surface-exchange mechanism that is mediated by high concentrations of these peptides^69^. We predicted peptides derived from H and F CDV proteins that could bind to MHC class II molecules (Fig. 1) to take advantage of their molecular properties in the context of MCH class II molecules (Table 2). Peptides that have entered outside the context of the native antigen must be subjected to a wide range of cell-surface and extracellular proteases throughout the immunization process, becoming an important limitation. Exogenous antigen and immunogenic peptides from potential vaccine candidates can enter the endosomal pathway by macropinocytosis or by receptor-mediated events, such as B-cell-surface-immunoglobulin uptake of antigen or Fc-receptor-mediated uptake of immune response complexes^70^. Once the immunogenic peptide enters the endocytic section, protease action must be deleterious; by the cathepsin family, proteins are in charge of antigen degradation^69^. Then, the immunogenic peptides must overcome this challenge by surviving this environment and being transported to the MHC class II-rich endosomal compartment, where peptides are loaded onto the MHC class II molecules, a process led by chaperones responsible for removing invariant chains^70^, which has the advantage of involving peptides predicted to interact with MHC class II molecules (Table 2).

Overall, several studies have discussed the usage and utility of new subunit vaccines based on either single immunogenic peptides or multiepitope polypeptides^17,26,44,45,71^. In this study, we evaluated in silico and in vitro a multiepitope polypeptide that includes all potential immunogenic predicted single peptides that overcome all in silico and in vitro validations (Fig. 6A). The constructed multiepitope CDV polypeptide exhibited several in silico safe characteristics, such as being nonallergenic, nontoxic and having no homologous proteins in the canine proteome, and it has immunogenic potential since both the VaxiJen and IEDB tools provided favorable results, indicating that it can trigger a potent immune response without generating any desired allergenic or toxic reactions (Fig. 6B). On the other hand, we also evaluated the cytotoxicity of the polypeptide in Vero-Dog-SLAM and MDCK cell lines and in primary cells, PBMCs and cRBCs and obtained considerable positive results for a potential polypeptide-based vaccine (Fig. 6C,D). Therefore, multiepitope vaccines may be recognized as a promising platform therapy against viral infections, with in silico safety and immunogenicity evaluations^17^. One possible concern is the immune response, which is robust enough to be protective as a vaccine formulation. However, other studies on SARS-CoV-2^72^ and this study have included different epitopes as single peptides or multiepitope polypeptides to facilitate both cellular and humoral immune responses.

Recently, the development of new-generation vaccines for viral pathogens such as CDV has advanced since vaccine failure was reported^9,73^. This vaccine failure has been explained through the emergence of diverse CDV strains, which must also be led not only by evolution but also by vaccination, which has played an important role in CDV lineage variation^74^. Moreover, lineage-specific neutralizing epitopes from the CDV H protein of diverse lineages, different from those of vaccine-based Onderstepoort strains, have been reported^75^. Understanding the genetic variation of CDV may become even more critical over time if the low protection afforded by available vaccines becomes more predominant around the world^76^. Although there are some new vaccine candidates based on recombinant platforms and other nonconventional vaccine alternatives^77^, there is an essential need not only to understand the transmission dynamics of CDV^78^ but also to improve and develop vaccines for nondomestic species^76^. Some alternatives have emerged as experimental bivalent vaccines employing vectors such as replication-defective human adenoviruses that express the CDV H protein and rabies proteins or bacterium-like particles that express the CDV H and F proteins^79,80^. Regardless of the effort of vaccine alternatives in animals, there are still limitations in the design of veterinary vaccines based on the available tools used to predict potential immunogenic molecules, such as peptides, in humans; however, the methodology employed in this study has been suggested^81,82^, indicating the importance of continuing to explore new approaches in the field of veterinary vaccines and vaccinomics^20^. These facts invite us to reflect on the necessity of finding a way to develop an ideal universal vaccine suitable for domestic and nondomestic animals threatened by CDV and other viral agents in the context of One Health.

## Methods

### CDV H and F protein consensus sequence analysis

Sequences from H and F proteins of CDV variants reported worldwide were obtained from the NCBI database. Consensus sequences were generated for both the H and F CDV proteins with EMBOSS^83^, to predict peptides based on the consensus sequences of the H and F CDV proteins.

### Prediction of potential immunogenic peptides based on CDV H and F protein sequences and peptide selection

Potential immunogenic peptide prediction employing H and F protein consensus sequences was carried out with different computational tools to predict helper CD4+ T cells, CD8+ T cytotoxic cells, and linear B-cell epitopes. To accomplish this goal, the online tools MHC2PRED (http://crdd.osdd.net/raghava/mhc2pred/), CTLPRED (http://crdd.osdd.net/raghava/ctlpred/), IEDB from the La Jolla Institute (https://www.iedb.org/), and SVMTRIP (http://sysbio.unl.edu/SVMTriP/) were used to predict the immunogenic peptides derived from the consensus sequences of the H and F CDV proteins for CD4+ and CD8+ T-cell and B-cell linear epitopes. All these computational tools are based on support vector machines that enable the prediction of diverse peptides based on databases that have been trained with peptides that have positive and negative desired functions.

### Antigenicity, allergenicity, toxicity potential, and physicochemical property evaluation

In silico safety was determined for selected peptides derived from CDV H and F consensus sequences. Antigenicity, the allergenic profile, and toxicity prediction were characterized for individual predicted peptides using the VaxiJen v2.0 server (http://www.ddg-pharmfac.net/vaxijen/), AllerTOP v2.0 server (https://www.ddg-pharmfac.net/AllerTOP/), and ToxinPred server (http://crdd.osdd.net/raghava/toxinpred/). The VaxiJen v2.0 tool enables the determination of whether a peptide sequence has the potential to be a viral antigen^84^. The AllerTop v2.0 server uses a k-nearest neighbor (kNN) method, amino acid descriptors, and ACC transformation methods to isolate nonallergens from allergens with 85.3% prediction accuracy via fivefold cross-validation^85^. The ToxinPred server estimates the properties of different peptides by employing support vector machines (SVMs), a machine learning approach with a quantitative matrix for predicting toxicity^86,87^. The ProtParam tool from ExPASy was used to measure the physicochemical properties of the peptides by the Swiss Institute of Bioinformatics^88^.

### BLAST homology assessment of CDV-derived peptides

NCBI Protein BLAST (BLASTp) was used to determine the homology between the selected peptides and the canine-reported proteome. The purpose of this cross-checking analysis was to avoid the inclusion of self-protein peptides in the dog proteome. To determine dog homology, the BLAST (https://blast.ncbi.nlm.nih.gov/Blast.cgi) tool protein BLAST module was used. In this experiment, comparisons were made with the default parameters for *Canis lupus familiaris* (taxid: 9606), and the threshold e-value was set to 0.05. Immunogenic peptides were determined to be nonhomologous peptides when no hits under the threshold e-value were found^89,90^.

### Homology modeling and validation of canine MHC-I or -II, TLR-2, and TLR-4

Homology models were constructed to evaluate the interactions between the selected peptides and canine MHC-I, MHC-II, TLR-2, and TLR-4; since no crystallographic structures were available, homology modeling was performed. The amino acid sequences of canine MHC-I, MHC-II, TLR-2, and TLR-4 were retrieved from the NCBI. Human proteins were used as templates (PDB Codes: 5F1N, 4FQXA, 4FQXB, 6NIG, 4G8A). MODELLER v. 10.0 was used to model the 3D structure. A total of 100 different structures with model quality scores (molpdf, DOPE, GA341) were obtained^91^. The homology models and the respective templates were overlapped to determine the root mean square deviation (RMSD) differences between the model and template structures by employing TM-Align, a protein structure alignment algorithm based on the TM-score^92^. The homology models were validated with bioinformatics tools such as SWISS-MODELTM, which provides global and local model quality based on the Z score and QMEAN, and each residue quality calculation from the amino acid sequence and Ramachandran plots were used to establish amino acids in energetically favorable regions, regarding dihedral angles ψ against φ of amino acid residues in the protein structure^51^. ProSA-Web, a Z score for the overall model quality tool, was used to check whether the Z score value of the input structure was within the range of scores typically found for native proteins of similar size, with the PDB as the reference database^93^.

### Molecular docking and dynamics of CDV peptides and canine MHC-I or MHC-II, TLR-2, and TLR-4

The interactions between the selected peptides and MHC-I, MHC-II, TLR-2, and MHC-4 were assayed by employing three online molecular docking tools. HPEPDOCK is a blind protein-peptide docking tool that functions through a hierarchical algorithm. Instead of running lengthy simulations to refine peptide conformations, this tool considers peptide flexibility through an ensemble of peptide conformations generated by the MODPEP program^94^; MDOCKPEP is a server that predicts ab initio protein–peptide complex structures starting with the protein structure and peptide sequence in three steps^95^; and CABSDOCK is a server that provides an interface for modeling protein–peptide interactions using a highly efficient protocol for the flexible docking of peptides to proteins^96^. For all the molecular docking tools, the ligand and the receptor are considered rigid structures. The most likely poses were selected based on the best tool scores. Molecular dynamics simulations were carried out employing GROMACS® software (Groningen Machine for Chemical Simulation, developed at the University of Groningen, The Netherlands)^97^. A topology file of the protein–peptide complexes was generated, and the conditions of the water box were established in a neutral ionic environment. The system was equilibrated, and the simulations were conducted with a constant number of molecules, temperature, and pressure (NVT and TPN assemblies). The final molecular dynamics simulation was run for 50 ns for each complex, and the trajectories were analyzed with Xmgrace software (Oregon Graduate Institute of Science and Technology, Hillsboro, OR, USA). All 3D complex graphics were generated using the software UCSF Chimera^98^.

### Peptide synthesis and cytotoxicity in cell lines and cPBMCs

Peptides were synthesized by BIOMATIK (USA) using standard solid-phase synthesis with a purity > 75% and characterized by mass spectrometry. The Vero cell line expressing dog signaling lymphocyte activation molecules (named Vero-Dog-SLAM), which was kindly donated by Yusuke Yanagi from Kyushu University, Fukuoka, Japan, and the MDCK cell line (Madin-Darby canine kidney) from the ATCC were cultured in Dulbecco’s modified Eagle’s medium (DMEM) supplemented with 2% fetal bovine serum (FBS) (Gibco, Grand Island, NY, USA) and 1% antibiotic/antifungal at 37 °C in a humidified atmosphere containing 5% CO_2_. Cell monolayers from the cell lines mentioned above were harvested, seeded in 96-well plates, and inoculated after 24 h. Then, the cells were treated with each peptide diluted in DMEM supplemented with 2% FBS (Gibco, Grand Island, NY, USA) at twofold serial dilutions from 6.125 to 200 nM for 72 h. For cPBMCs, peripheral blood from two healthy donors was diluted with 1× PBS at a 1:1 ratio; then, a density gradient was generated by adding 3 mL of Ficoll-Histopaque 1077 (Sigma-Aldrich, St. Louis, MO, USA) and centrifuging for 23 min at 2300 rpm. The interface that corresponded to the PBMCs was recovered, and the cells were washed with 1× PBS and centrifuged at 1800 rpm for 5 min. Then, the PBMCs were treated with each peptide diluted in RPMI supplemented with 10% FBS (Gibco, Grand Island, NY, USA) at twofold serial dilutions from 6.125 to 200 nM for 72 h. Afterward, the cells were washed twice with PBS, and 50 μL of MTT solution (to a final concentration of 0.5 mg/mL) was added to each well and incubated for 2 h at 37 °C. The formazan precipitates were dissolved with the addition of 100 μL of DMSO, and the absorbance at 450 nm was measured using a microplate reader (Thermo Fisher Scientific, USA). Wells without peptide treatment were used as a viability negative control, while wells treated with 0.5% Triton X-100® served as a cytotoxicity positive control. Two independent experiments with 4 replicates (n = 8) were carried out for each cell line, and nontreated cells were used as negative controls. Additionally, peptides Y, B, and K1, which were kindly donated by Sergio Orduz and have reported antimicrobial activity potential^99^, were used as noncytotoxic control peptides. The percent viability was determined based on the absorbance of the nontreated cells. Means and standard deviations are reported.

### Ethical considerations

This study received approval from the Ethics Committee for Animal Experimentation at the Universidad Cooperativa de Colombia in Bucaramanga. All procedures adhered to pertinent guidelines and regulations. Written informed consent was obtained from the legal guardians of all animals participating in this research.

### Hemolytic potential of the peptides in canine red blood cells

The hemolytic potential of the selected peptides was established by using canine red blood cells (cRBCs) from fresh canine blood obtained from two healthy donors and collected in sodium citrate buffer. Informed consent was obtained from the participants’ legal guardians. Blood samples were centrifuged at 1000×*g* for 7 min at room temperature. One milliliter of the pellet was washed three times with 3 mL of PBS, and the red blood cells were suspended in 9 mL of PBS. Afterward, cRBCs were treated with twofold serial dilutions from 6.125 to 200 nM in a U-bottom 96-well plate and incubated for 3 h at 37 °C and 90 rpm. The samples were centrifuged at 1000×*g* for 7 min, and 50 μL of the supernatant from each peptide treatment was placed in another flat-bottom 96-well plate. The absorbance at 620 nm was measured using a microplate reader. Two independent experiments with 4 replicates (n = 8) were performed. PBS was used as a negative hemolytic control, and 0.1% Triton X-100® was used as a positive hemolytic control. The percent hemolysis was calculated as the difference between the treatment and control absorbance.

### Multiple-epitope polypeptide construction and in silico and in vitro evaluation

A multiepitope polypeptide was constructed based on the best single peptides identified via in silico and in vitro assays. We employed amino acid likers such as AYY, GPGPG, and KK for the MHC-I, MHC-II and B-cell epitopes, respectively^17^. Polypeptide was synthesized by BIOMATIK (USA) using standard solid-phase synthesis with a purity > 95% and characterized by mass spectrometry. Similar in silico and in vitro validation through viral antigenicity in VaxiJen v2.0 and IEDB immunogenicity, tools, allergenicity, toxicity potential, potential homologous canine peptides, canine TLR-2 or TLR-4 polypeptide interaction, and cytotoxicity and hemolytic assays were carried out at concentrations ranging from 6.125 to 25 nM, respectively, as described above for single peptides.

### Patents

J.R.S. and S.R.M. had a patent PCT/IB2022/061236 pending.

## Conclusions

The use of computational tools and immunoinformatic approaches has enabled the design and development of rational next-generation vaccines. In this study, we constructed a peptide library and selected a group of immunogenic peptides based on the main antigenic determinant proteins H and F from CDV, and 12 peptides were selected. Although they showed significant in silico results, in silico and in vitro safety protocols were used to evaluate the antigenicity, toxicity, and allergenicity of these peptides and their cytotoxicity in cell lines and primary cells, in addition to their hemolytic potential when evaluated as single peptides and multiepitope polypeptides. The development of peptide-based vaccines represents a promising avenue in the field of vaccinology. Through rational design and incorporation of immunogenic epitopes, peptide vaccines offer several advantages, including safety, specificity, and ease of production. Continued research efforts aimed at optimizing peptide vaccine formulations, delivery systems, and adjuvants are imperative to unlock their full therapeutic potential. We anticipate that our prediction model will exhibit positive effects in vivo to prevent CDV infection in domestic and wild animals since there is a need to formulate a universal CDV vaccine in which multiepitope polypeptide vaccines, including genetic information, are available for all linages circulating worldwide.

### Supplementary Information

Supplementary Information.

## Data Availability

The datasets analyzed during the current study are available from the institutional repository at 10.57924/7J5SKQ.
