# Supplementary material for: Universal peptide-based potential vaccine design against canine distemper virus (CDV) using a vaccinomic approach
Source: Sci Rep. 2024 Jul 18;14:16605. doi: 10.1038/s41598-024-67781-5 (PMC11258135; doi:10.1038/s41598-024-67781-5)
Supplement: Supplementary file 1 — Supplementary Information. [file 41598_2024_67781_MOESM1_ESM.docx]

**Universal peptide-based potential vaccine design against canine distemper virus (CDV) using a vaccinomic approach with an integrated *in silico* and *in vitro* validation**

**Santiago Rendon-Marin^a,b^, Julián Ruíz-Saenz^a,*^**

**^a^Grupo de Investigación en Ciencias Animales - GRICA, Facultad de Medicina Veterinaria y Zootecnia, Universidad Cooperativa de Colombia, sede Bucaramanga**

**^b^Corporación Ciencias Básicas Biomédicas Universidad de Antioquia, Medellín, Colombia**

**Supplementary Figures and Tables**


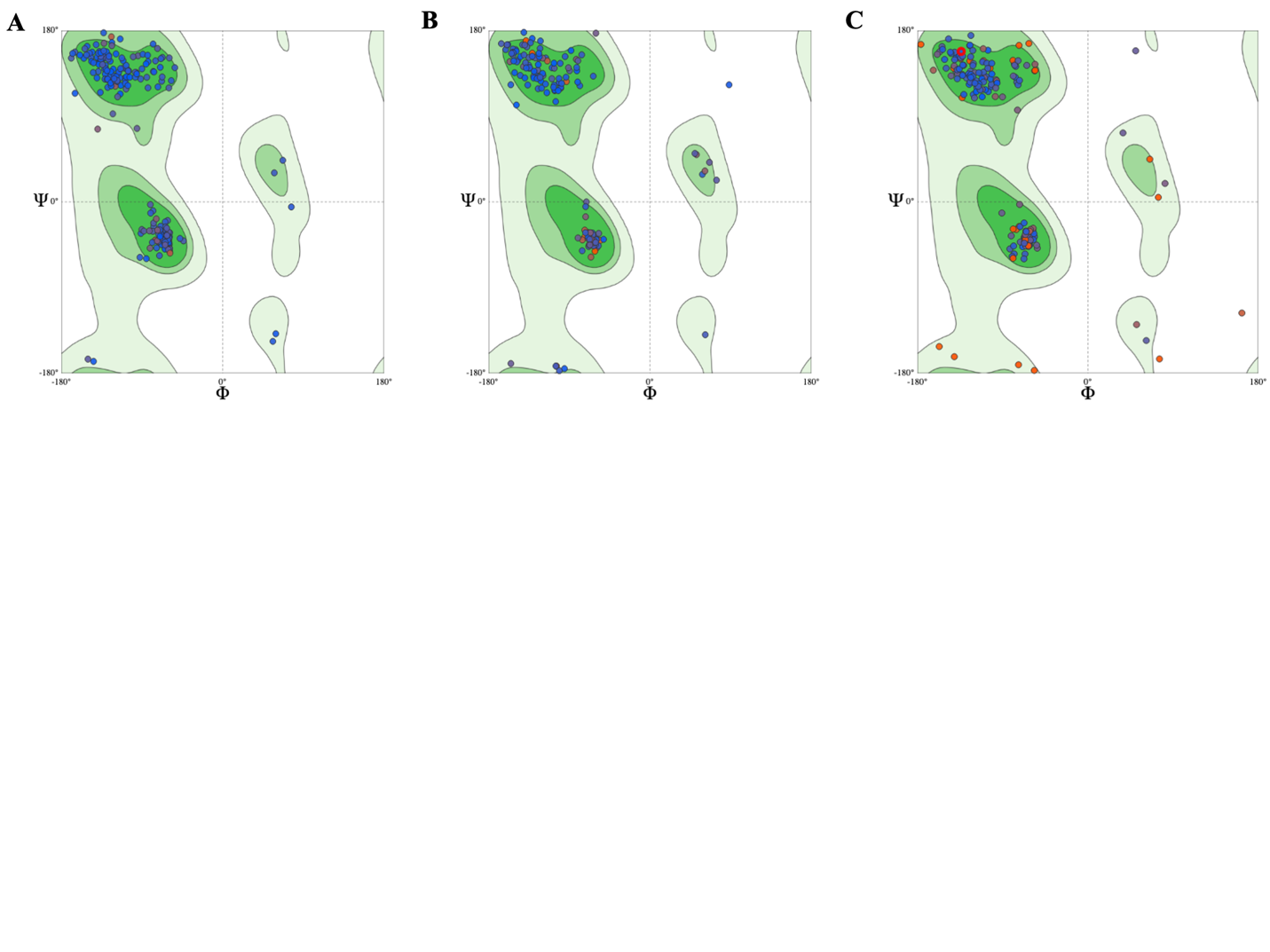


**Supplementary Figure 1**. Ramachandran plot of canine protein models. A) DLA-I-88. B) DLA-II-Alpha. C) DLA-II-Beta. These plots were created with SWISS-MODEL (https://swissmodel.expasy.org/).


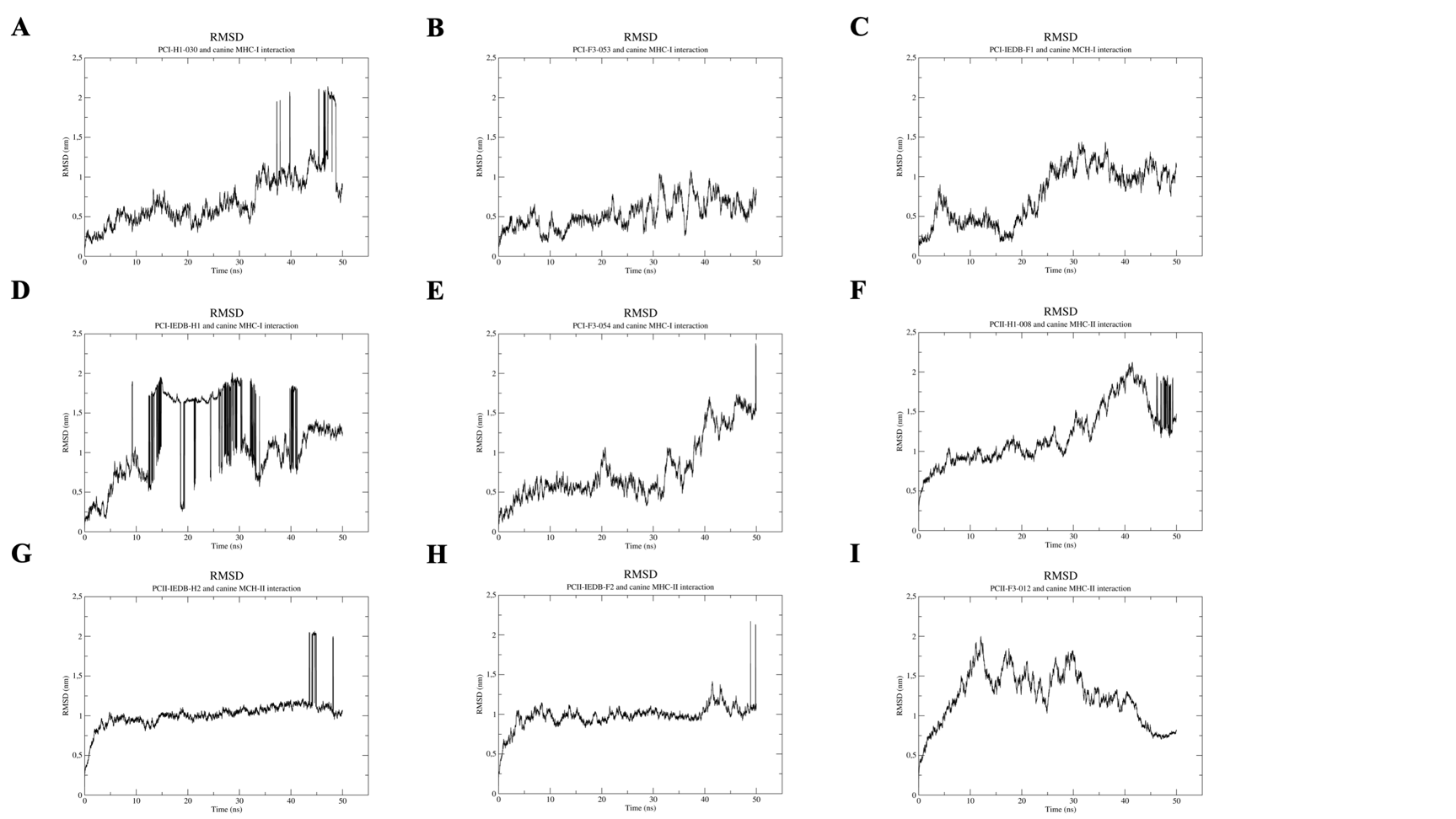


**Supplementary Figure 2**. Molecular dynamic simulations of selected peptides docked with canine MHC molecules. Root mean square deviation (RMSD) plot of: A) PCI-H1-030 and MHC-I. B) PCI-F3-053 and MHC-I. C) PCI-IEDB-F1. D) PCI-IEDB-H1 and MHC-I. E) PCI-F3-054 and MHC-I. F) PCII-H1-008 and MHC-II G). PCII-IEDB-H2 and MHC-II. H) PCII-IEDB-F2 and MHC-II. I) PCII-F3-012 and MHC-II. Graphs for molecular dynamics were obtained with Xmgrace software (Oregon Graduate Institute of Science and Technology, Hillsboro, OR, USA).

**Supplementary Table 1. Validation data of all models obtained by homology modelling.**

| **Protein** | **Z Value** | **Favorable region (%)** | **TM Value** | **Align AA** |
| --- | --- | --- | --- | --- |
| **DLA-I-88** | -9.4 | 98.16 | 0.99066 | 274 |
| **DLA-II-Alpha** | -5.34 | 96.00 | 0.88193 | 177 |
| **DLA-II-Beta** | -5.27 | 94.33 | 0.89979 | 194 |
| **5F1N** | -9.19 | 95.60 | - | - |
| **4FQX-Alpha** | -5.47 | 99.44 | - | - |
| **4FQX-Beta** | -5.33 | 96.17 | - | - |

*5F1N, 4FQX alpha and beta are the MHC molecules templates reported in the PDB

**Supplementary Table 2. Protein BLAST homology assessment of selected peptides with *Canis lupus familiaris* proteome.**

| **ID** | **Peptide** | **Length** | **Protein** | **Coverage (%)** | **Identity (%)** |
| --- | --- | --- | --- | --- | --- |
| **P1** | **PCI-H1-004** | 9 | Sodium-dependent noradrenaline transporter | 100 | 80 |
| **P2** | **PCI-H1-030** | 11 | N-terminal kinase-like protein | 63 | 100 |
| **P3** | **PCII-H1-008** | 9 | DNA polymerase epsilon catalytic subunit A | 100 | 75 |
| **P4** | **PCI-F3-053** | 12 | Prosalusin | 91 | 81,8 |
| **P5** | **PCII-F3-012*** | 9 | Terminal uridylyltransferase 4 | 100 | 85,7 |
| **P6** | **PCI-IEDB-H1** | 9 | Pecanex-like protein 1 | 77 | 85,7 |
| **P7** | **LINB-IEDB-H** | 11 | Cat eye syndrome critical region protein 2 | 81 | 100 |
| **P8** | **PCI-F3-054** | 12 | Cornifin-B-like | 75 | 56.3 |
| **P9** | **PCII-IEDB-H2** | 13 | FERM domain-containing protein 5 | 76 | 80 |
| **P10** | **PCI-IEDB-F1** | 9 | BCLAF1 and THRAP3 family member 3 | 55 | 100 |
| **P11** | **PCII-IEDB-F2** | 13 | Carboxypeptidase D | 92 | 53 |
| **P12** | **LINB-IEDB-F** | 9 | Vacuolar protein sorting-associated protein 13D | 100 | 75 |

*****This peptide has the same sequence than P5.1, however, it was predicted to be presented in both MHC class I and II.
